# Solar to fuels conversion technologies: a perspective

**DOI:** 10.1007/s40243-017-0088-2

**Published:** 2017-01-30

**Authors:** Harry L. Tuller

**Affiliations:** 1 Department of Materials Science and Engineering, Massachusetts Institute of Technology and Materials Processing Center, Cambridge, MA 02139 USA; 2 International Institute of Carbon Neutral Energy Research, Kyushu University, Fukuoka, Japan

**Keywords:** Solar fuels, CO_2_ sequestration, Hydrogen economy, Photoelectrochemistry, Electrolysis, Fuel cell, Thermochemical processes, Solar energy

## Abstract

To meet increasing energy needs, while limiting greenhouse gas emissions over the coming decades, power capacity on a large scale will need to be provided from renewable sources, with solar expected to play a central role. While the focus to date has been on electricity generation via photovoltaic (PV) cells, electricity production currently accounts for only about one-third of total primary energy consumption. As a consequence, solar-to-fuel conversion will need to play an increasingly important role and, thereby, satisfy the need to replace high energy density fossil fuels with cleaner alternatives that remain easy to transport and store. The solar refinery concept (Herron et al. in Energy Environ Sci 8:126–157, 2015), in which captured solar radiation provides energy in the form of heat, electricity or photons, used to convert the basic chemical feedstocks CO_2_ and H_2_O into fuels, is reviewed as are the key conversion processes based on (1) combined PV and electrolysis, (2) photoelectrochemically driven electrolysis and (3) thermochemical processes, all focused on initially converting H_2_O and CO_2_ to H_2_ and CO. Recent advances, as well as remaining challenges, associated with solar-to-fuel conversion are discussed, as is the need for an intensive research and development effort to bring such processes to scale.

## Introduction

Fossil fuels are broadly used for transportation, electricity generation, industrial processes, and heating. Given their ready availability, high energy density,^1^ and ease of handling, storage, and transport, they supply more than 80% of the world’s overall energy needs, and 96% of the transportation sector’s energy demand, with much of the remaining 4% of transportation energy being electricity generated in plants that burn fossil fuels. At the same time, the combustion of fossil fuels to extract their stored chemical energy is a major source of greenhouse gas emissions, mostly carbon dioxide (CO_2_), and thus contributes to global warming. While interest in and utilization of solar energy as a key alternative clean energy source have grown rapidly in recent years, solar technology deployment has been largely directed to electricity generation. While important, recent advances in solar electricity generation do not address the continued need for high energy density fuels for transportation, heating, and industrial process uses, which together account for roughly 70% of overall energy requirements. This report discusses options for converting solar energy into fuels, largely through the solar-driven conversion of water and carbon dioxide into fuels and chemicals. This conversion would be achieved in a *solar refinery* [1], where solar energy acts on CO_2_ captured from flue gas emissions, together with water, to generate *solar fuels*. These fuels, which can be sustainably produced in liquid or gaseous form, offer multiple benefits in terms of grid stability, energy security, compatibility with existing infrastructure, and climate change mitigation. The opportunities and challenges associated with sourcing, producing, storing, and distributing solar fuels are the focus of this report.^2^

## Solar alternative fuels

Increasingly, electricity, including the widespread electrification of transportation, is being seen as playing a pivotal role in achieving deep cuts in greenhouse gas emissions, such as the reductions—at 80% below 1990 levels—proposed for California by 2050 [2]. Even more aggressive goals recently articulated by the White House aim to derive 80% of electricity from clean energy sources by 2035 and reduce greenhouse gas emissions 17% by 2020 and 83% by 2050 (relative to a 2005 baseline) [3]. Given that electricity accounts for 30% of global energy consumption, and without an unexpected breakthrough in electricity storage,^3^ alternative, low-carbon fuels will be needed to satisfy the remaining 70% of global energy requirements, particularly for transportation, manufacturing, and heating [4]. To get a sense of the magnitude of this challenge, one need only note that the U.S. registered light-duty vehicle (LDV) fleet of over 234 million vehicles consumes 8.4 million barrels of oil to travel 7.3 billion miles on a daily basis. This represents nearly 10% of total petroleum liquids consumption worldwide [5–8].

Solar energy, among all carbon-free energy sources, is viewed by some experts, as the alternative with the greatest intermediate to long-term potential to replace fossil fuels [9]. For this to happen, however, two important challenges must be addressed. The first is providing adequate *energy storage capabilities* for solar-generated electricity, given the intermittent character of the solar resource. The second, perhaps more important challenge, is utilizing solar energy to aid in the *production of clean alternative fuels* for the transportation, industrial, and housing sectors [10].

Solar energy has, until now, accounted for a relatively small fraction of the overall energy supply, with its fluctuating contributions to the grid controlled and compensated by thermal generation (fossil-fuel combustion). As solar and wind penetration increases, however, the intermittency of these two energy sources seriously compromises the stability and quality of grid power. This issue has already begun to demand urgent attention in Germany where 36.8 terawatt-hours (TWh) of electricity (equal to 6.6% of total production)^4^ was generated by solar sources in 2015 [11]. During this same period, 87.1 TWh (equal to 15.6% of Germany’s total electricity production) was generated by nuclear plants, which are slated for shut down by 2021 [12]. While dependence on intermittent renewable energy sources is not yet quite so high in the United States, where solar and wind accounted for 0.6 and 4.7% of overall electricity production in 2015, respectively [13], the relentless decline in PV module prices has continued at a rate of 5–7% annually for the past decade [14]. The U.S. Energy Information Administration now expects utility-scale solar generating capacity to increase by more than 60% between the end of 2014 and the end of 2016, while wind capacity is expected to increase by about 23% over the same time period [15]. While these projections may be optimistic, and may assume the continued existence of various government subsidies, there is little doubt that generation from these intermittent energy sources will continue to show significant growth. This creates a strong incentive to bring into play, as quickly as possible, alternative storage systems that are both robust and carbon–neutral to ensure grid stability in the coming decades.

A figure-of-merit for the storage of electrical energy generated by intermittent sources, defined as the ratio of the value of stored electricity to the cost of storage, is useful in comparing alternate storage technologies. For example, assuming a 1-day storage period, the figure-of-merit for electrical energy stored chemically via hydrogen produced by electrolysis is 12.7. While this is much higher—taking into account the cost, life, and efficiency of the process—than electrical battery storage, which has a figure-of-merit of 1.0 [16], these figures do not account for the efficiency of producing hydrogen or converting hydrogen back to electricity. If one combines the efficiency of electrolysis cells, at approximately 75%, with that of a combined cycle gas and steam turbine generator running on hydrogen (about 60%), the result is a full-cycle electricity–fuel–electricity efficiency of up to 45% [17]. More typical round trip efficiencies are reportedly closer to 30%, making the attraction of hydrogen vs battery storage less clear in the short term [18]. Hydrocarbon fuels with higher energy densities can also be synthesized by combining hydrogen with CO_2_ captured, for example, from coal-burning plants. Longer term, as fossil-fuel generating plants are replaced by renewable sources, CO_2_ could be captured from non-combustion sources such as cement plants.

Siemens reports that synthetic natural gas (i.e., methane [CH_4_]) can be generated, on a pilot scale, from hydrogen and CO_2_ with up to 80% efficiency [17]. Synthetic natural gas has three times the energy density, on a volume basis, of hydrogen. Given the central role that chemical fuels already play in electricity generation, the conversion of solar energy into chemical fuels that are capable of being used in the existing distribution and end-use infrastructure would be highly desirable [9]. *Synthetic gas*, stored and distributed like conventional natural gas, could then be used to power vehicles or in heating systems—in addition to being used to generate electricity—on an as-needed basis. In Germany, for example, existing natural gas storage capacity—at more than 200 TWh—would be sufficient to satisfy consumption for several months [19]. Moreover, synthetic hydrocarbons can be used in a variety of additional ways, including in the production of fertilizer, plastics, and pharmaceuticals, as well as for transportation and heating [20].

Like most other countries, the United States is nearly completely dependent on petroleum for transportation; in fact, petroleum use for transportation accounts for about one-third of total annual U.S. CO_2_ emissions [21]. Worldwide, the transportation sector accounted for 19% of global energy demand in 2012 and oil supplied 96% of this demand, with the rest coming from natural gas, biofuels, and electricity [22]. Government regulations mandating improved vehicle fuel efficiency and the increasing electrification of transportation via the introduction of hybrid and plug-in vehicles will help reduce dependence on fossil fuels. However, *many forms of transportation, including long*-*haul passenger vehicles, ships, trucks, and aircraft, will continue to require high energy density, but ideally carbon*-*free or carbon*-*neutral fuels.*

### Basic solar fuels

**Fig. 1:**
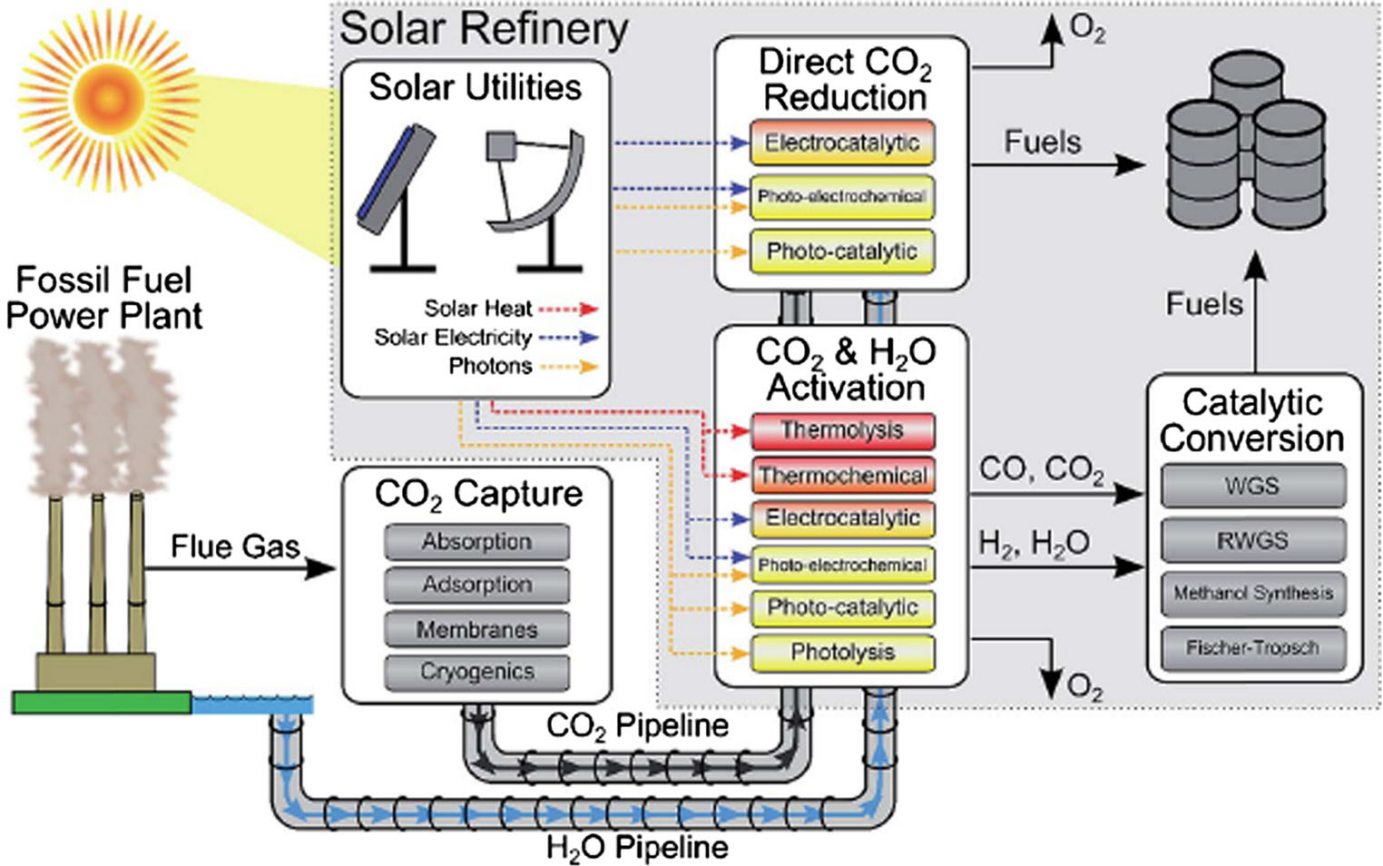
Schematic of a Solar Refinery and solar fuel feedstocks (CO_2_, H_2_O, and solar energy) captured onsite or transported to the refinery. The Solar Utility provides energy in the form of heat, electricity or photons used to convert the CO_2_ and H_2_O into fuels either by direct CO_2_ reduction or solar activation of CO_2_/H_2_O to CO/H_2_ and subsequent catalytic conversion to fuels (e.g., via methanol synthesis or by the Fischer–Tropsch method. Color code: yellow—ambient; red—elevated temperatures (from Herron et al. [1])

Solar fuels are not new. The photo-assisted synthesis (photosynthesis) of chemical fuels, in the form of plant matter, is fundamental to life on Earth and supports all current biomass. The same process, over geological time, produced the fossil fuels on which human civilization has depended for the vast majority of its energy needs for the past century and earlier. Due to the relative inefficiency of natural photosynthesis, the use of all cultivatable land on Earth to produce biofuels would not satisfy humanity’s projected energy needs in the coming decades, particularly if one takes into account the energy needed to harvest, store, distribute, and convert biomass into useful chemical fuels [23]. An alternative approach that obviates the need to set aside vast tracts of arable land is to replicate the essential elements of photosynthesis found in natural organisms with artificial systems. On an industrial scale, one can visualize a *solar refinery* (see Fig. 1) that converts readily available sources of carbon and hydrogen, in the form of CO_2_ and water (H_2_O), to useful fuels, such as methanol (CH_3_OH), using energy sourced from a *solar utility* [1]. The solar utility, optimized to collect and concentrate solar energy and/or convert solar energy to electricity or heat, can be used to drive the electro-catalytic, photoelectrochemical, or thermochemical reactions needed for conversion processes. For example, electricity provided by PV cells can be used to generate hydrogen electrochemically from water via an electrolysis (electrocatalytic) cell.

Hydrogen, the most elemental fuel, has many attractive attributes—it is clean burning (water being the only by product of hydrogen combustion) and can be efficiently converted back to electricity via fuel cells. However, hydrogen lacks volumetric energy density, and cannot be easily stored and distributed like hydrocarbon fuels. Rather than utilizing solar-generated hydrogen directly and primarily as a fuel, its utility is much greater—at least in the short to intermediate term^5^—as an onsite fuel for converting CO_2_ to CH_4_ or for generating syngas, heat, or electricity [24]. Reacting CO_2_ with hydrogen (H_2_) not only provides an effective means for storing CO_2_ (in methane, for example), but also produces a fuel that is much easier to store, distribute, and utilize within the existing energy supply infrastructure. Thus, recycling CO_2_ to produce a hydrocarbon fuel would open the transportation sector to far greater reliance on renewable energy beyond what is currently feasible with rechargeable electric vehicles (at present, such vehicles comprise fewer than 3% of all vehicles sold in the United States) [8]. The idea of converting CO_2_, a product of combustion, to useful hydrocarbon fuels by harnessing solar energy is attractive in concept. However, significant reductions in CO_2_ capture costs and significant improvements in the efficiency with which solar energy is used to drive chemical conversions must be achieved to make the *solar refinery* a reality. We address these issues in greater detail below.

Solar energy collected and concentrated within a solar utility (see Fig. 1) can be harnessed in different ways: (1) PV systems could convert sunlight into electricity which, in turn, could be used to drive electrochemical (electrolysis) cells that decompose inert chemical species such as H_2_O or CO_2_ into useful fuels; (2) *photoelectrochemical* or *photocatalytic* systems could be designed wherein electrochemical decomposition reactions (like the reactions in the previous example) are driven directly by light, without the need to separately generate electricity; and (3) *photothermal* systems could be used either to heat working fluids or help drive desired chemical reactions such as those connected with thermolysis, thermochemical cycles, etc. (see Fig. 1). Each of these approaches can, in principle, be used to generate environmentally friendly *solar fuels* that offer “efficient production, sufficient energy density, and flexible conversion into heat, electrical, or mechanical energy [25].” The energy stored in the chemical bonds of a solar fuel could be released via reaction with an oxidizer, typically air, either electrochemically (e.g., in fuel cells) or by combustion, as is usually the case with fossil fuels. Of the three approaches listed here, only the first (PV and electrolysis cells) can rely on infrastructure that is already installed today at a scale that would have the potential to significantly affect current energy needs. The photoelectrochemical and photothermal approaches, though they hold promise for achieving simplified assembly and/or high energy conversion efficiencies, require considerable development before moving from the laboratory into pilot scale and commercially viable assemblies. Remaining sections of this report discuss the status of these three approaches and the challenges that must be overcome to advance each of them.

Given that, the contribution of artificially produced solar fuels, such as hydrogen and methane, remains extremely small at present, exceptional efforts—particularly to reduce costs—are needed to bring these clean and sustainable fuels up to meaningful levels. Critical challenges that will need to be overcome include improving the sourcing and collection of CO_2_, increasing the efficiency of solar-assisted catalytic conversion of CO_2_ and H_2_O into fuels, extending device lifetimes, reducing costs, and investing in infrastructure upgrades to reduce the large gap between current laboratory demonstrations and deployable technology. These challenges are discussed here in terms of key candidate solar fuels.

Chapters 2 and 3 of the MIT *Future of Solar Energy* study [26] present a detailed analysis of options for generating electricity from sunlight via PV cells. A few highlights from that analysis are worth repeating here by way of providing context for this report. According to the Solar Electric Power Association, installed solar power generating capability in the United States totaled 10.7 GW as of 2013. PV accounted for most of this capability, roughly half of which (48%) was provided by utility-scale installations. Another 25 GW of solar generation capacity is projected to be installed by 2017 [27]. *Solar thermal power*, also referred to as *concentrated solar power* (*CSP*), represents a growing but much smaller component of installed solar power generating capacity. As of 2013, 926 MW of CSP capacity had been installed in the United States, but an additional 800 MW was anticipated in 2014 with a total of 3.2 GW of capacity projected by 2017 [27]. While generally more costly than PV, CSP enables lower cost thermal (rather than electrical) energy storage, which is key to overcoming issues related to solar energy’s intermittency. It is worth noting that of the approximately 1.06 TW of electrical power capacity in the United States in 2012 [28], 10.7 GW of solar power would represent only 1% of the total.

#### Hydrogen production

Hydrogen has been recognized for some time as providing a potential foundation for a clean, flexible, and secure energy future. The fact that it is accessible in the form of water makes hydrogen highly attractive. When hydrogen is used as a fuel, either by combustion or electrochemically in a fuel cell, the only byproduct is water—a feature that promises an emission-free environment. While the combustion of hydrogen produces more energy on a mass basis (39.5 kWh/kg)^6^ than the combustion of any other fuel—e.g., 2.4 and 2.8 times the energy of methane and gasoline combustion, respectively [29]—hydrogen has low energy density by volume. In fact, at 2.8 kWh per liter, the energy density of hydrogen is 3.5 times lower than that of gasoline. Since liquefying hydrogen is highly energy intensive, and thus not practical, hydrogen is most effectively stored as a gas in high-pressure tanks. Given its simple chemical structure, it is one of a very small group of fuels capable of being used in low-temperature fuel cells, thereby making it the fuel of choice for fuel cell-powered vehicles [30].

Since hydrogen in its molecular form does not occur in nature, it is not an energy source and must be produced. In this sense, hydrogen is rather more like electricity, a convenient energy carrier, and, as will become evident, a strong synergy exists between electricity, hydrogen, and other renewable energy sources [31]. Hydrogen production today is actually a large net *generator* of CO_2_ emissions, with 13.7 kilograms (kg) of CO_2_ produced for every kg of H_2_, on average [32]. At present, approximately 96% of hydrogen is derived from fossil fuels and only 4% is produced via electrolysis [33]. Hydrogen is produced in high volumes (current global annual production exceeds 70 million metric tons, while annual U.S. production is projected to total 11 million metric tons in 2016) [34], largely via steam reforming of natural gas (methane)^7^ for use in fertilizers and for use in the hydrocracking of heavy petroleum and the manufacture of methanol and hydrochloric acid. The value of hydrogen production worldwide is expected to reach $163 billion by 2015 [34, 35]. Hydrogen produced via water electrolysis is generally more expensive than by large-scale fuel processing techniques, although it becomes more attractive when produced onsite. However, if fossil fuels are used to generate the electricity that drives the electrolysis process, resulting emissions are actually higher than for natural gas reforming [36]. *This points to the need and opportunity to harness renewable sources of energy, particularly intermittent sources such as solar and wind, for hydrogen production.* The next sections review the two main options being considered for generating hydrogen using solar energy.

##### Water electrolysis

Water (H_2_O) can be decomposed into its elemental components hydrogen (H_2_) and oxygen (O_2_) by passing current between two electrodes immersed in an electrolysis cell. Oxygen is evolved at the positive electrode (anode), hydrogen is evolved at the negative electrode (cathode), and the two are separated from each other by an ionic conducting liquid or solid electrolyte that selectively transports H^+^, OH^−^ or O^2−^ ions across the cell, depending on the nature of the electrolysis cell. A cell voltage of at least 1.23 V is required. In practice, however, voltages closer to 1.9 V are needed to achieve reasonable current densities, and corresponding fluxes of generated hydrogen and oxygen gases. The need for higher voltage to overcome ohmic (resistive) losses and electrode over-potentials in turn reduces electrical-to-chemical energy conversion efficiencies. There is thus great interest in identifying and optimizing catalysts that can accelerate the oxygen oxidation reaction at the anode and the hydrogen reduction reaction at the cathode, thereby reducing electrode over-potentials.

Two approaches to harnessing solar energy to drive the electrolysis reaction are possible and are being pursued. The most obvious approach is to drive a conventional water electrolyzer using the electrical output of PV devices. Given that typical conversion efficiencies are 11.5–17.5% for commercial PV systems and 63–73% for electrolyzers, overall conversion efficiencies of approximately 12% can be expected and have been reported for optimized, combined PV–electrolyzer systems [1, 37]. The most obvious advantage of this approach is that both PV and electrolysis systems are commercially available, although large-scale electrolysis systems are not nearly as extensively available as PV systems.

An alternative approach, still in the experimental stage, is the use of photoelectrolytic systems that combine the functions of light collection, charge separation, and electrolysis in a single cell. This is achieved by replacing one or both of the metallic electrodes in a conventional electrolysis cell with a semiconductor. The advantage of this approach is that it offers opportunities to minimize cost by eliminating redundant support structures and energy losses associated with cell interconnections. At the same time, it has been difficult to simultaneously achieve high conversion efficiencies and long-term operating stability, given that the semiconductors offering the highest efficiencies are susceptible to corrosion during cell operation. Several recent advances address these limitations. These include: (1) combining the PV and electrolysis cells into a single integrated tandem photoelectrochemical (PEC) cell, with theoretical solar-to-hydrogen conversion efficiencies of 31.1% at one Sun illumination [38], (2) protecting the semiconductors in PEC cells from corrosion [39], (3) introducing low-cost Earth-abundant catalysts [40], and (4) improving active area and optical absorptivity through the use of nanostructuring or nanowires [41]. These options are described in the next sections.

##### Combined PV-electrolysis systems

The MIT *Future of Solar Energy* study [26] methodically compares the principal PV materials [e.g., crystalline and amorphous silicon (Si), cadmium telluride (CdTe), gallium arsenide (GaAs) and copper indium gallium diselenide (CIGS)] and device designs in terms of their relative costs, solar to electricity conversion efficiencies, long-term stability, and environmental impact. Overall, it is fair to say that a number of PV systems are now commercially available, with the option to trade off lifetime costs and efficiency depending on the particular application being considered. That being the case, it is more useful here to review options for electrolyzers that are generally less advanced in their development and commercialization.

Water electrolysis is a relatively mature technology, with hydrogen production capacities ranging from a few cubic centimeters per minute (cm^3^/min) to thousands of cubic meters per hour (m^3^/h). Key performance parameters for electrolyzer systems are conversion efficiency [electrical to chemical energy (H_2_)]; current density (amps/unit area), which in turn determines the hydrogen flux density, durability, scalability, and cost; and, for some designs, reliance on noble metals such as platinum. The three major types of electrolyzers are based on aqueous alkaline (OH^−^), solid polymer (H^+^), and solid oxide (O^2−^) ionic conducting electrolytes. The most commercially developed option is the *alkaline cell*, which uses a 30% potassium hydroxide electrolyte solution that operates at 80–90 °C and pressures to 25–30 bar. Cathodes and anodes are porous nickel (Ni) coated, respectively, with platinum at the cathode and with metal oxides at the anode. Such cells exhibit good durability (10–20 years) and have efficiencies of 63–73%, but suffer from relatively low current densities, which means that larger systems are required to produce equivalent volumes of hydrogen [1, 31]. The proton exchange membrane (PEM) electrolyzer, which uses a polymer-based Nafion proton conducting membrane and has a working temperature of about 90 °C, can operate at considerably higher current densities than the alkaline cell and, therefore, can be more compact, but its conversion efficiency is lower—on the order of 56%. The PEM cell also suffers from reliance on precious metal electrocatalysts (typically platinum dispersed on carbon), a costly membrane (Nafion), and potential degradation in performance due to catalyst coarsening that reduces the active electrode area over time. The solid oxide electrolyzer cell utilizes a ceramic oxygen ion conducting electrolyte [typically yttria stabilized zirconia (YSZ) or Y_0.1_Zr_0.9_O_2_], and operates at much higher temperatures (500–850 °C) and at pressures of 30 bar. The higher operating temperature allows for a significant reduction in electrical power consumption (efficiencies as high as 85–90% have been reported) and for the use of non-noble metal electrodes (typically Ni-YSZ cermet cathode and a ceramic lanthanum strontium manganese oxide (LSMO) anode) [42]. Reduced ohmic and over-potential losses also allow for considerably higher current densities and more compact designs. The high operating temperatures and brittle nature of the oxide components, however, create additional challenges vis-a-vis reduced lifetimes and materials and fabrication costs. The key features of these electrolysis systems are summarized in Table 1, which is taken from Herron et al. [1] Table 1 and includes estimates of projected solar-to-H_2_ conversion efficiencies, which range from 8.5% for PEM to 12% for solid oxide electrolysis cells, assuming a 15% solar-to-electricity PV conversion efficiency.

**Table 1:** Summary of solar-driver water splitting technologies (from Herron et al. [1])

System	Operation conditions		System efficiency	Solar-to-H_2_ efficiency (%)	Advantages	Disadvantages
	*P* (bar)	*T* (°C)				
Alkaline electrolysis	25–30	80–90	63–73%	10^a^	Commercial technology low capital cost	Low current density H_2_–O_2_ mixing
PEM electrolysis	<85	<100	56%	8.5^a^	High current density Compact design H_2_–O_2_ produced separately	High capital cost for membrane Precious metal catalyst
Solid oxide electrolysis	30	500–350	85–90%	12^a,b^	High electrical efficiency Non-noble catalyst H_2_–O_2_ produced separately	Brittle ceramics
Photo-electrochemical	1	25	–	12	High solar efficiency H_2_–O_2_ produced separately	Degradation
Photo-catalytic	1	25	–	0.2	Simple process	H_2_–O_2_ are mixed Low solar efficiency
Thermolysis	1	2,200	–	1–2	Simple process	Low materials stability H_2_–O_2_: are mixed High radiative losses
Thermochemical	–	>700	40%	18	High energy efficiency H_2_ –O_2_ produced separately	High capital cost Complex process design

^a^Assuming 15% solar PV efficiency

^b^Does not account for thermal energy

^c^Assuming 45% solar to thermal efficiency

To increase the energy efficiency of electrolysis, the cell voltage must be reduced (efficiency ~1.23 V/cell operating voltage). This in turn requires better catalysts or a decrease in current density. Reductions in current density, however, translate to a reduction in the rate of hydrogen production, which tends to increase required electrode area and thus cost [43]. Thus, catalyst development remains a key target in nearly all electrochemical devices. Furthermore, besides the basic cell-stack, the so-called balance-of-plant components, i.e., power supply/voltage regulator, water supply and circulation, gas separators, heat exchanger, controls and instrumentation, add significant costs to these systems. These balance-of-plant costs should be kept in mind when considering the relative attractiveness of alternative devices based on the direct integration of the PV and electrochemical functions in photoelectrochemical systems as discussed in the following section.

**Fig. 2:**
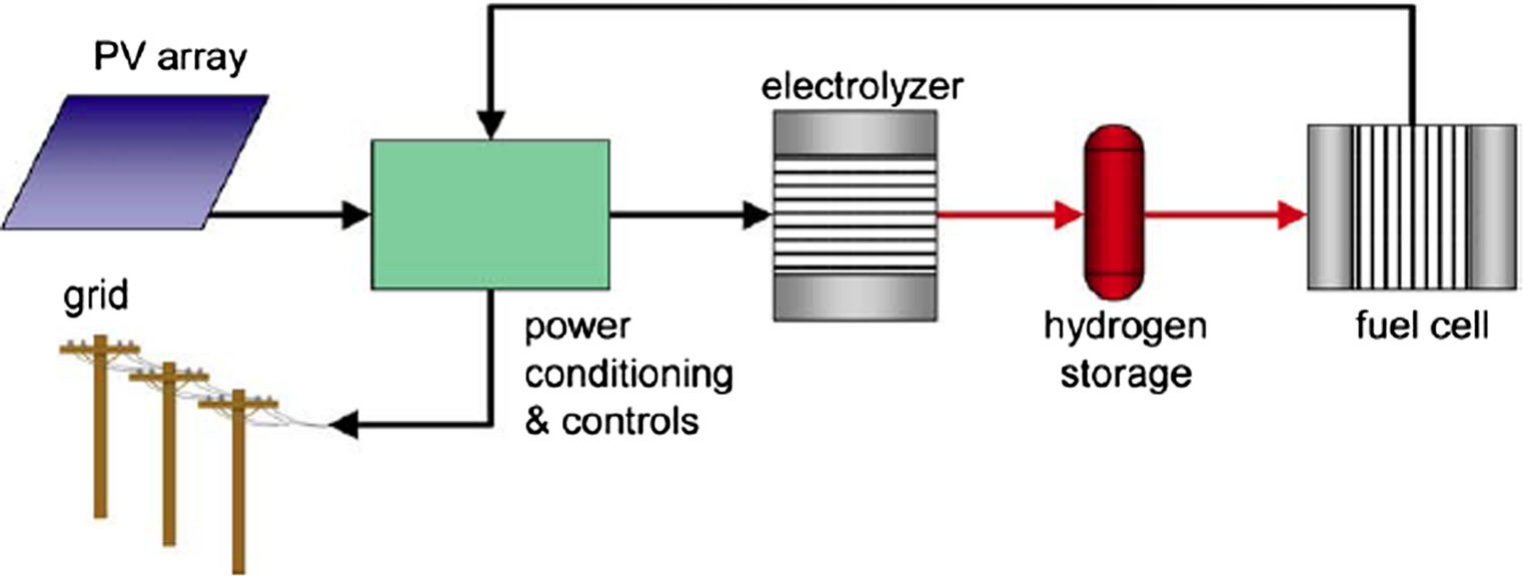
Schematic diagram of integrated PV-hydrogen utility energy system (from Ref. [31])

Barbir [31] has considered a number of alternative applications in which PV arrays might be coupled with PEM electrolyzers for grid-independent hydrogen generation or joint grid-electricity and/or hydrogen generation, with and without storage. In general, to match the electrolyzer’s voltage–current requirements to the variable power output of the PV system, a dc/dc power regulator must be part of the power conditioning and controls system. To enable the electrolyzer to operate at its optimum design point, a tie-in with the grid helps eliminate problems with intermittent electrolyzer operation by combining PV output with electricity inputs from the grid. To deliver a required load profile to the grid, a regenerative fuel cell (combination of electrolyzer and fuel cell with hydrogen storage) is added, as illustrated in Fig. 2. The power conditioning and control unit directs power from the PV array to either the grid or the electrolyzer, switches to fuel cell power when there is insufficient power from the PV array, and provides voltage regulation for both electrolyzer and fuel cell. The regenerative fuel cell system is reportedly less costly than battery storage for high power, long duration storage, although both approaches at this time remain costly [44].

Given the relatively rapid drop in PV costs in recent years, one might expect that electrolyzer costs would be the limiting factor in combined PV–electrolysis cells. Instead, a recent techno-economic analysis by Rodriguez et al. [45] demonstrates that the cost of hydrogen is largely defined by the PV component (which accounts for up to 97% of the total cost) while materials selection for the electrolysis cell has only minor effects. This finding follows from the fact that the area of the PV array may need to be more than 100 times that of the electrolysis cell given the much lower current densities (<10 mA/cm^−2^) in unconcentrated solar cells as compared to electrolyzers, which can operate at current densities above 1 A cm^−2^. As a consequence, PV cells operating with solar concentrators can be expected to lead to considerable cost savings. The authors estimate that optimized systems can achieve costs below $2.90 per kg of hydrogen produced, including compression and distribution costs [45]. This compares favorably with the U.S. Department of Energy’s stated goal of reducing the cost of hydrogen production to $2.00–$4.00 per gallon of gasoline equivalent (gge)^8^ delivered and dispensed by 2020 [30].

##### Photoelectrochemical water splitting

**Fig. 3:**
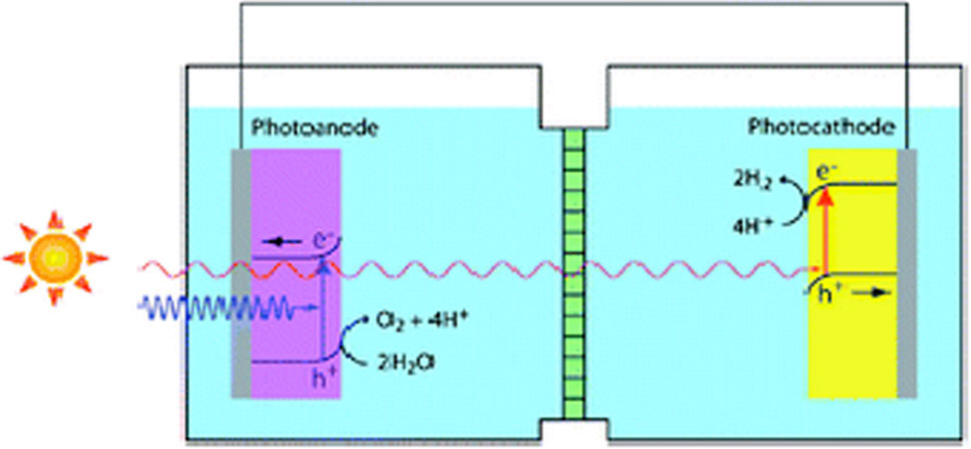
A schematic of a two photoelectrode photoelectrochemical cell in which the n-type photoanode and p-type photocathode are selected so that the low energy photons not absorbed by the photoanode are absorbed by the photocathode (from Ref. [46])

A *photoelectrolysis* cell, illustrated in Fig. 3, is inherently more attractive since it combines the functions of PV cells and conventional electrolysis cells in a single unit. Light (photons) absorbed in a photoelectrode create electron–hole pairs that are separated by internal electric fields, as in PV cells. After separation, the holes drive the respective water oxidation reaction (forming O_2_) at the photoanode and electrons drive the water reduction reaction (forming H_2_) at the photocathode. Similar cells can be designed with only a photocathode or photoanode with the counter electrode typically being made of platinum. Key challenges to overcome include relatively low solar-to-hydrogen conversion efficiencies (typically under 5%), and the high costs and photo-assisted corrosion of the covalently bonded semiconducting photoelectrodes that support higher conversion efficiencies. Semiconducting oxides are typically more resistant to photo-corrosion and are composed of non-toxic, Earth-abundant elements, but their higher band gap energies limit the absorption of a significant fraction of the incident solar radiation at longer wavelengths and are less efficient at separating the photo-generated electrons and holes and at driving the oxygen and hydrogen generation reactions at the electrode–liquid interfaces.

**Fig. 4:**
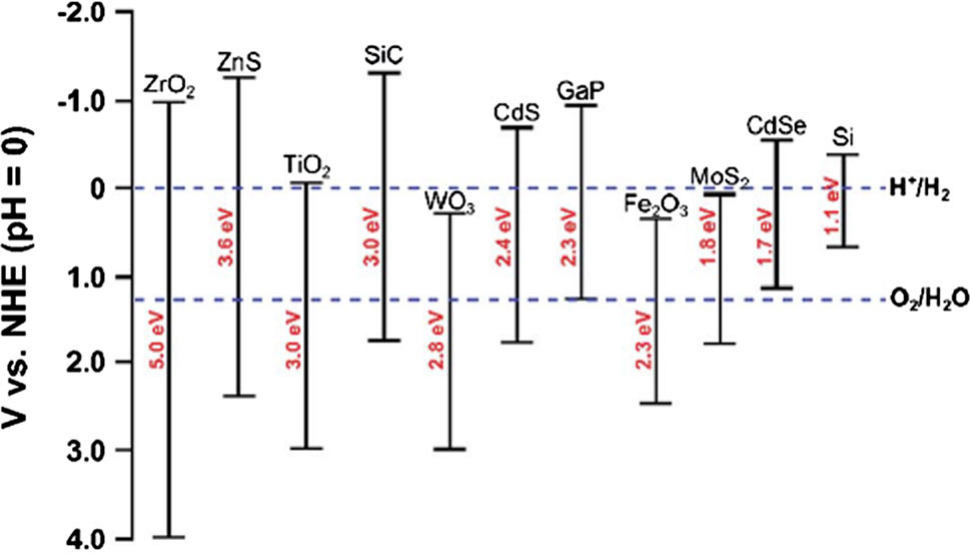
Conduction and valence band edges and band gaps for a series of common oxide and covalently bonded semiconductors relative to the redox potentials for hydrogen and oxygen evolution reactions (from Herron et al. [1])

Often the assistance of an external bias or voltage is needed due to the poor alignment of the energy bands in the solid with the redox levels in solution. Figure 4 makes clear why the number of photoelectrode semiconductor candidates is much more restricted than for PV systems. A key criterion for the solar absorber is that its band gap energy be of such magnitude [~1.4 electron volts (eV)] that it absorbs a significant fraction of the incident infrared and visible sunlight without losses to heat from absorption of higher energy photons with energies much above the band gap energy. As a photoelectrode, not only must the band gap be of the correct magnitude, but the band edges must straddle the redox potentials for hydrogen and oxygen evolution reactions (see Fig. 4). Taking titanium dioxide (TiO_2_) as an example, its conduction band barely straddles the redox potential for hydrogen evolution and its band gap of 3.0 eV falls in the ultraviolet range of the spectrum, which leaves only several percent of the incident solar radiation that can be absorbed by the material. The result is a solar-to-hydrogen conversion efficiency of only about 0.4% [47]. As in the conventional electrolysis cell, while only 1.23 V is thermodynamically required to split water at room temperature, losses—largely due to electrode over-potentials—increase the minimum required voltage to about 1.9–2.0 V [48]. As is evident from Fig. 4, iron (III) oxide (Fe_2_O_3_), a highly Earth-abundant and low-cost material, has a somewhat larger-than-optimal band gap and nearly straddles both the hydrogen and oxygen redox potentials. As a consequence, this material, also known by its mineral name *hematite,* has been receiving a great deal of attention [49] (see also additional references below). Challenges for Fe_2_O_3_, as for many larger band gap oxides, include poor charge transfer (extremely short minority carrier diffusion lengths) and large electrochemical over-potentials.

In recent years, significant progress has been achieved in improving the performance of hematite and other oxide semiconductors by increasing the exposed active areas of these devices through nanostructuring [50], introducing improved oxidation catalysts to minimize over-potentials [51, 52], modifying the doping profiles of the semiconductors to enhance electron–hole separation [53], using dye sensitization [54] and light-trapping cavities to enhance light absorption [55], and improving control of the defect structure [56]. If cell efficiencies can be increased to 15% and if cell life can be extended to 25 years, the cost of hydrogen would be lower than that generated by PV cells in concert with electrolyzers [29].

**Fig. 5:**
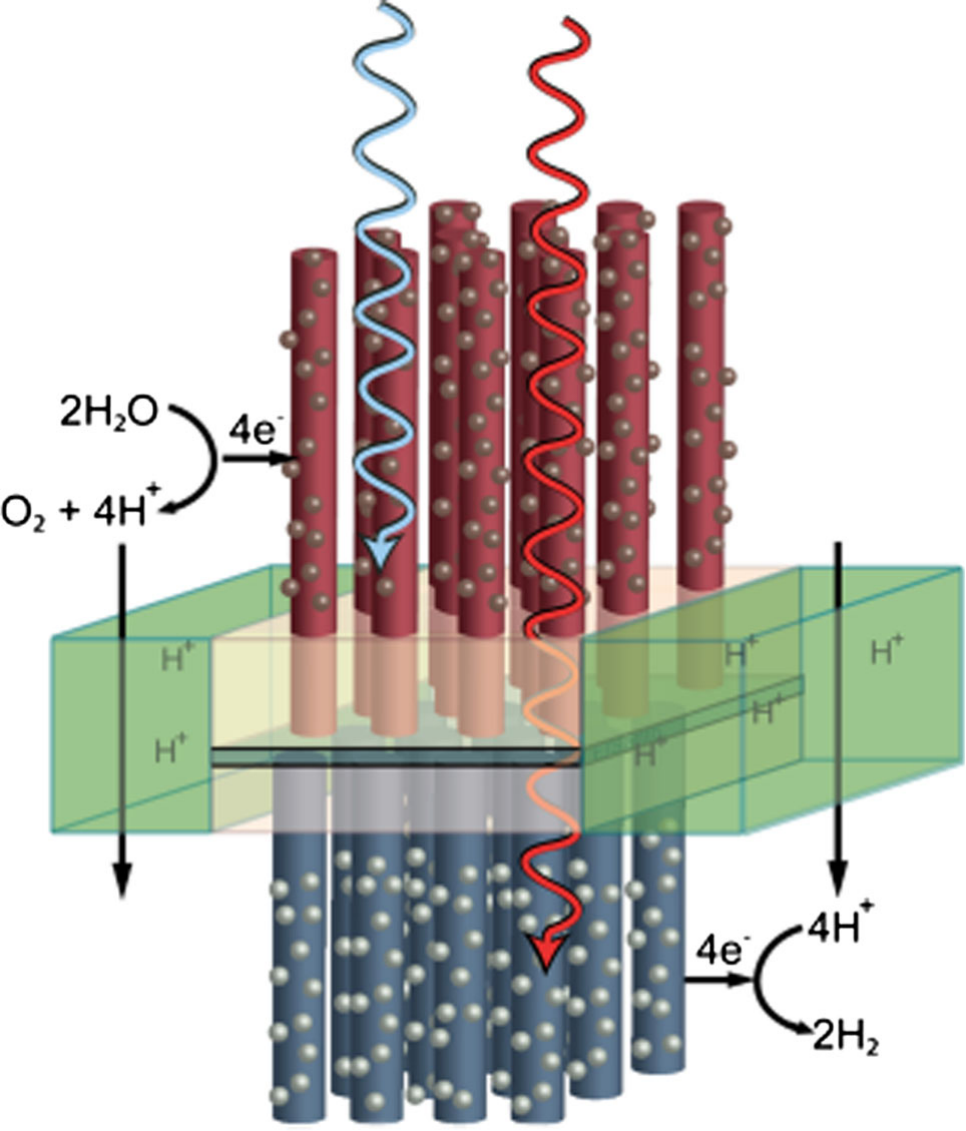
Illustration of a PEC cell with photocathodes and photoanodes in nanowire forest configuration with the anodic and cathodic chambers separated by a proton permeable membrane. Light capture is enhanced by having the nanowires oriented parallel to the incident radiation, while minority charge carriers need only diffuse short distances along the radii of the wires to reach the solution interface. From Lewis group [62, 63]

Other efforts have been directed toward replicating cells in which both cathode and anode are photo-active and in which photons that are not absorbed by the smaller band gap semiconductor (e.g., the photoanode) are absorbed by the larger band gap semiconductor (e.g., photocathode), as illustrated in Fig. 3. Such tandem designs enable the use of smaller band gap (i.e., 1.1–1.7 eV) light absorbers that are well matched to the solar spectrum, while simultaneously providing the necessary photovoltage required to electrolyze water (that is, the voltage exceeds the sum of the thermodynamically required potential [1.23 V], resistive losses, and over-potentials required to drive the water splitting reactions at anode and cathode at a given current density [57]). The use of a tandem structure also relaxes the cell’s stability requirements, thereby enabling the use of photocathodes that are stable under cathodic (but not necessarily anodic) conditions, and vice versa for the photoanodes [58]. In other tandem designs, the oxygen evolution and hydrogen evolution reactions occur at electrocatalysts (e.g., Pt) deposited on and driven by tandem semiconducting *light absorbers* that covert the incident light into a photovoltage [57]. Early examples developed at the National Renewable Energy Laboratory (NREL) were composed of a p-type GaInP_2_ PEC cell connected to a GaAs PV cell and exhibited solar-to-hydrogen conversion efficiencies of 12%, but they suffered from rapid corrosion [59]. More recent results have been obtained for silicon-hematite (Si/Fe_2_O_3_) multijunction photoanodes in which Earth-abundant silicon (Si) acts as the photo-absorber and iron oxide acts as the catalyst. Cells of this type have produced current densities as high as 17 mA cm^−2^ [60]. A similar arrangement combining an Si photo-absorber with an n-type tungsten trioxide photoanode showed considerably lower current densities, but demonstrated the use of a silicon micro-wire forest electrode array that offers orthogonalization of light absorption and charge-carrier collection [61]. As illustrated in Fig. 5, light is absorbed along the length of the wires, allowing the use of low-cost semiconductors, such as Si, characterized by a more weakly absorbing indirect band gap. At the same time, minority holes in the photoanode or minority electrons in the photocathode need only diffuse a short distance along the radius of the wire to reach the solid–liquid interface, thereby enabling the use of easily grown, low-minority carrier diffusion-length materials. Tandem structures, in which Si is paired with 1.6–1.8 eV band gap semiconductors, promise solar-to-hydrogen efficiencies greater than 25% [57]. Key parameters include identifying solar absorbers with high fill factors while matched to achieve high photocurrent densities, highly efficient electrocatalysts, and low electrolyte ohmic resistance [57].

##### Solar thermochemical hydrogen production

*Solar thermal power*, or *CSP*, is an alternate means of harnessing incident solar energy that can be utilized to generate solar fuels, in addition to its more common use, in which high temperatures generated by concentrating incident solar radiation are used to drive conventional steam or gas turbines [64]. Several routes to using CSP for solar fuel generation are discussed in the following. While thermochemical approaches are particularly promising, progress to date has been largely limited to laboratory demonstrations.

**Fig. 6:**
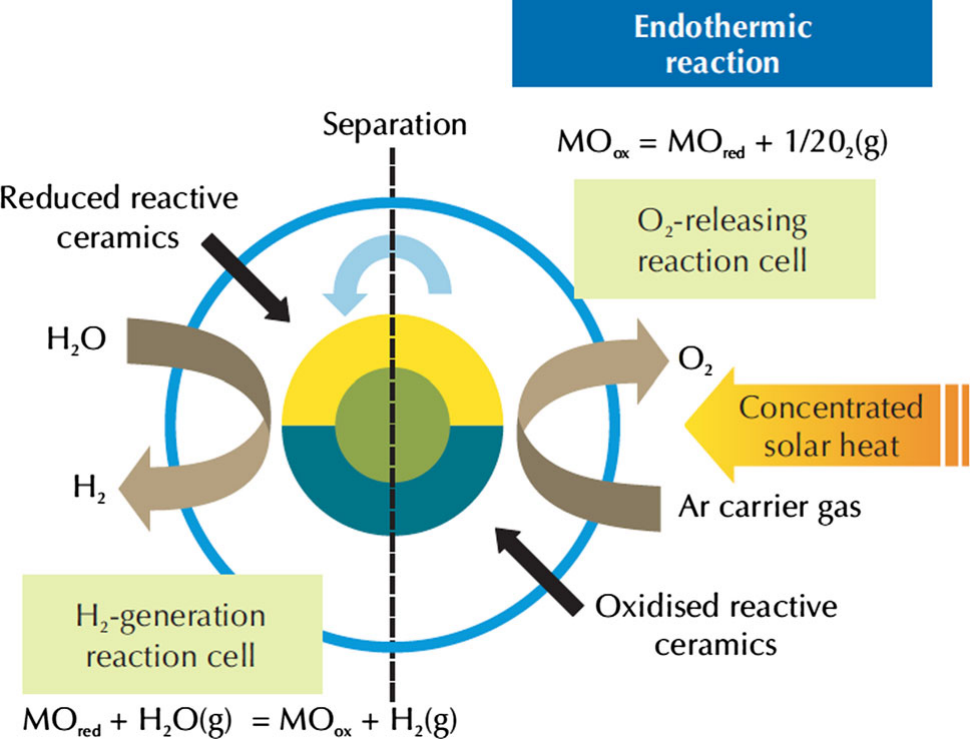
Solar-driven, two-step water splitting to form hydrogen based on reduction/oxidation reactions [70]

Heating water to sufficiently high temperatures to cause water molecules to split into hydrogen and oxygen or cause methane molecules to split into hydrogen and carbon by *solar thermolysis* requires temperatures above 2200 °C. This approach is not being actively pursued given the difficulty of reaching such high temperatures by solar concentration and given the fact that very few containment materials can stand up to these extremes. On the other hand, high-temperature, solar-driven, *thermochemical* fuel production, based on the ability to induce low-cost metal oxides to release oxygen (i.e., to reduce) by heating them to high but more moderate temperatures (typically 500–1500 °C), with the aid of concentrated solar energy technology, is being investigated. These oxygen-deficient materials are then subsequently exposed to water or CO_2_ at a lower temperature, which allows them to recover their lost oxygen, thereby releasing hydrogen from water or CO from CO_2_ (see Fig. 6). This two-step process eliminates the need for high-temperature gas separation of, for example, the H_2_ and O_2_ formed during water thermolysis, and allows coordination with the daily solar cycle (the reduction step can occur during daylight, the re-oxidation step during the evening). Alternatively, the two steps can be separated spatially by delivering the deoxidized materials where the hydrogen is needed, e.g., at refueling stations or chemical plants, thereby achieving higher volumetric energy densities than are available with compressed hydrogen. Recent examples of materials that have been studied for solar-driven, *thermochemical* fuel production include zinc oxide (ZnO) decomposition into Zn metal [65] or the reduction of cerium dioxide (CeO_2_) to its oxygen-deficient form, CeO_2-x_ [66]. Theoretical solar-to-fuel efficiencies as high as 35–50% have been estimated, but these assume high rates of fuel production and a high level of heat recovery [67]. A recent analysis suggests that a solar-to-methanol system that achieved 7.1% efficiency with H_2_O and CO_2_ as feedstocks would result in methanol’s price being competitive with that of other renewable-resource-based alternatives [68].

While the solar *thermochemical* process provides a clean, efficient, and sustainable route for producing hydrogen from water, challenges include reducing heat losses during each step of the process cycle, scaling up and improving the coupling of the solar concentrators to the reactors [69], identifying appropriate reactor containment materials and seals, and ensuring that the working materials continue to allow for rapid reduction and oxidation during the cycling process even after many thermochemical cycles.

#### Carbon dioxide-derived fuels

The earth is going through a rapid period of warming due to high levels of global CO_2_ emissions from human activity (34 billion tons in 2011 [71]). CO_2_ concentrations in the atmosphere are still low enough (0.04%) that it would be impractically expensive to capture and purify CO_2_ from the atmosphere. But other sources of CO_2_ are available that are considerably more concentrated. Power generation based on natural gas or coal combustion is responsible for the major fraction of global CO_2_ emissions (including 38% of overall emissions in the United States), with other important sources being represented by the cement, metals, oil refinery, and petrochemical industries [72]. Nearly, 8000 large, stationary sources of CO_2_ exist worldwide, each with annual CO_2_ emissions above 0.1 Mt [73]. Indeed, a growing number of large-scale power plant *carbon dioxide capture and storage* (CSS) projects are either operating, under construction, or in the planning stage, some of them involving facilities as large as 1200 MW capacity [74, 75]. While solar PV energy conversion has the potential to reduce CO_2_ emissions by serving as an alternative means of generating electricity, harnessing solar energy to convert the CO_2_ generated by other sources into useful fuels and chemicals that can be readily integrated into existing storage and distribution systems would move us considerably closer to achieving a carbon–neutral energy environment. This section reviews options for CO_2_ capture and separation.

Herron et al. [1], in a very recent review, examine the main routes for CO_2_ capture from stationary sources with high CO_2_ concentrations derived from *post*-*combustion, pre*-*combustion, and oxy*-*combustion* processes. In *post*-*combustion,* flue gases formed by combustion of fossil fuels in air lead to gas streams with 3–20% CO_2_ in nitrogen, oxygen, and water. Other processes that produce even higher CO_2_ concentrations include *pre*-*combustion* in which CO_2_ is generated at concentrations of 15–40% at elevated pressure (15–40 bar) during H_2_ enrichment of synthesis gas (syngas) via a water–gas shift reaction (WGS—see Fig. 1) and *oxy*-*combustion* in which fuel is combusted in a mixture of O_2_ and CO_2_^−^ rather than air, leading to a product with 75–80% CO_2_. CO_2_ capture can be achieved by absorption using liquid solvents (wet-scrubbing) or solid adsorbents. In the former approach, physical solvents (e.g., methanol) are preferred for concentrated CO_2_ streams with high CO_2_ partial pressures, while chemical solvents [e.g., monoethanolamine (MEA)] are useful in low-pressure streams. Energy costs for MEA wet-scrubbing are reportedly as low as 0.37–0.51 MWh/ton CO_2_ with a loading capacity of 0.40 kg CO_2_ per kg MEA. Disadvantages of this process are the high energy cost for regenerating solvent, the cost to compress captured CO_2_ for transport and storage, and the low degradation temperature of MEA. Alternatives include membrane and cryogenic separation. With membranes, there is an inverse correlation between selectivity and permeability, so one must optimize between purity and separation rate. Cryogenic separation insures high purity at the expense of low yield and higher cost. Currently, MEA absorption is industrially practiced, but is limited in scale: 320–800 metric tons^9^ CO_2_/day (versus a CO_2_ generation rate of 12,000 metric tons per day for a 500 MW power plant). Scale-up would be required to satisfy the needs of a solar refinery. Alternatives, such as membranes, have relatively low capital costs, but require high partial pressures of CO_2_ and a costly compression step to achieve high selectivity and rates of separation.

While capturing CO_2_ and converting it to liquid fuels serves society’s greater good, the important question still to be resolved is “what are the incentives for power plants and other industrial sources to pursue this approach?” Indeed, since carbon capture reduces the efficiency of power generation, power plants with carbon capture will produce more CO_2_ emissions (per MWh) than a power plant that does not capture CO_2_. Therefore, the cost of transportation fuel produced with the aid of CO_2_ capture must also cover the incremental cost of the extra CO_2_ capture [76]. These costs must then be compared to the alternative costs associated with large-scale CO_2_ sequestration, the practicality of which also remains to be demonstrated. Finally, one also needs to consider the longer term rationale for converting CO_2_ to liquid fuels once fossil-fuel power plants cease to be major sources of CO_2_. Closed-cycle fuel combustion and capture of CO_2_ from, e.g., vehicle tailpipes, present a considerably greater technical and cost challenge than capture from concentrated stationary sources.

CO_2_ can be converted to fuels with renewable, solar-derived hydrogen and solar heat, as discussed above. For example, the reverse-water–gas shift reaction (RWGS):

$$ {\text{CO}}_{2} + {\text{H}}_{2} \to {\text{CO + H}}_{2} {\text{O}} $$

can be used, in concert with catalysts (copper-, iron-, or ceria-based systems), to convert CO_2_ and hydrogen to CO and water. The CO mixed with hydrogen produces syngas, which can be used to generate a variety of products, including methanol, or liquid hydrocarbons through Fischer–Tropsch synthesis [77]. Issues related to the thermal stability of the catalysts and the undesired formation of methane still need to be resolved. There are also direct routes for hydrogenating CO_2_ to make products including methanol, methane, and formic acid. Besides these hydrogenation routes, CO_2_ can also, in principle, be converted to fuels using direct solar energy through electro-catalytic, photo-electrochemical, and thermochemical reduction, although these approaches remain in very early stages of development.

##### Electrolysis

CO_2_ can be electrolytically reduced to fuels in a manner similar to water electrolysis with oxygen evolving at the anode and CO_2_ reduction occurring at the cathode. The product of the reduction depends on the electro-catalyst used and can include formic acid, formaldehyde, methanol, methane, or ethylene. The main challenges include high cell overpotentials, low faradaic efficiency, low current densities, and electrocatalyst deactivation [78]. Because the thermodynamic potential for CO_2_ reduction is similar to that of water splitting (1.23 V), the process results in low faradaic efficiency, given the competition to generate hydrogen. Only copper is able to reduce CO_2_ to hydrocarbons (i.e., methane, ethylene) with significant current densities at moderate over-potentials and reasonable faradaic efficiency.

More promising is high-temperature CO_2_ reduction by solid oxide electrolysis cells (SOEC) [42]. As discussed above, high-temperature operation decreases the electrical energy required to drive the reaction, while simultaneously accelerating electrode reaction kinetics. Efficient reduction of CO_2_ at 800 °C has been achieved with the perovskite oxide electrode La_0.8_Sr_0.2_Cr_0.5_Mn_0.5_O_3_ (LSCM) in combination with a Pd–ceria/YSZ co-catalyst. Of particular interest is high-temperature co-electrolysis of H_2_O and CO_2_ that produces syngas at the cathode and O_2_ at the anode. Graves et al. [42] proposed a CO_2_-to-fuels process involving co-electrolysis, calculating that the process could operate at 70% electricity-to-liquid-fuels efficiency. While perhaps overly optimistic, given the high cost of atmospheric CO_2_ capture, their findings point to high-temperature co-electrolysis as a technology that is deserving of continued attention. A prototype 40 kW SOEC is to be installed by Haldor Topsoe A/S, a major Danish company noted for its catalysis technology, for the production of synthesis gas as part of project to convert biomass- and wind-generated electricity into synthetic fuels [79].

##### Photoelectrochemical and thermochemical approaches

Both these approaches are considerably less developed than the SOEC approach discussed above and are at the stage of early laboratory-scale studies. As with water splitting, the main challenge for the *photo*-*electrochemical* approach is to identify suitable photocathodes that enable reduction with the aid of visible light irradiation. Other complications include limited solubility of CO_2_ in aqueous solutions as well as competition from hydrogen evolution. Approaches being investigated include the use of non-aqueous solvents [80] or the use of three-phase (solid/liquid/vapor) interfaces in which metal-mesh electrodes are partially immersed in solution while CO_2_ is supplied from the vapor phase [81].

The *thermochemical reduction* of a metal oxide can be followed by re-oxidation with CO_2_ as the oxidant rather than with water. For example, ZnO can first be reduced to Zn at 1600 °C with the aid of solar heating, then subsequently cooled to 360 °C, at which point the Zn can be reacted with CO_2_ to form ZnO and CO [82]. The Zn/ZnO thermochemical cycle has a theoretical maximum solar-to-chemical**-**energy conversion efficiency of 39%, but as with thermochemical reduction of water to hydrogen, major losses are associated with poor heat recovery [83]. A key drawback of this process is Zn volatility, which requires that Zn vapor be separated from O_2_. An alternative option is the thermochemical co-reaction of CO_2_ and H_2_O with CeO_2_ to produce CO, H_2_, and O_2_. The reaction cycle (repeated 500 times) begins with CeO_2_ being reduced at 1420–1640 °C, followed by oxidation with CO_2_ and H_2_O at 900 °C. While theoretical solar-to-fuel efficiencies as high as 16–19% have been predicted, only 0.8% efficiency has been achieved experimentally with heat loss being the main drawback [66].

CO_2_ can be converted to useful products with the aid of hydrogen following recognized industrial processes, i.e., reduction of CO_2_ to CO using renewable solar hydrogen, syngas production by combining H_2_ with CO, and direct hydrogenation of CO_2_ to chemicals and other fuels. The result is a fuel that is easier to store, distribute, and utilize within the present infrastructure, as compared to hydrogen gas. Alternatively, CO_2_ can be directly reduced to fuel through PV-electrolytic or PEC methods. When compared to water splitting, however, conversion rates, efficiencies, and selectivity are low. Considerable work is needed to identify more efficient processes and catalysts for CO_2_ reduction by these more direct methods.

## Key challenges and opportunities

To meet increasing energy needs, while limiting greenhouse gas emissions over the next 20 years, an estimated 5–10 TW of power capacity from renewable sources will be needed. Given the limited ability of non-solar renewable resources (geothermal, wind, hydro, etc.) to supply energy on this scale, it is estimated that in the period 2030–2050, 10–25% of the world’s energy will need to come from solar energy [84]. A high fraction of solar investment will likely be directed to electrical energy generation given that the cost of solar electricity is approaching grid parity, at least in regions with high electricity rates. However, since electricity production currently accounts for only about one-third of total primary energy consumption, solar-to fuel conversion will need to play an increasingly important role.

As is evident from the above discussion, hydrogen can be expected to retain its central role as a solar fuel, given its broad utility in fuel cells, its potential role in coping with intermittent renewable generators, and its use as a basic feedstock chemical. However, hydrogen does have drawbacks, at least for the time being, with respect to storage and transport. Taking a broader perspective, hydrocarbon fuels that could be derived from direct conversion of CO_2_ and water by solar means and that are compatible with the existing energy and transportation infrastructure are highly attractive alternatives. However, the pathway to efficient, low-cost CO_2_-derived solar fuels must first overcome many technical challenges. More generally, rapid adoption of alternative energy conversion and storage technologies requires that costs be brought down to competitive levels.

Given the many possible options for reducing CO_2_ emissions via utilization of solar-assisted hydrogen production, it is a useful exercise to consider and rank these opportunities [85]. The first option is to use solar H_2_ to displace steam methane reforming as a means to generate H_2_ for use in fertilizer production and fuel refinery operations. This would require only a few percent of current annual global solar PV electricity generation, but it would have a noticeable impact on CO_2_ emissions. Similarly, solar hydrogen could be used to minimize the extent of water gas shift reaction in coal-to-chemical conversion plants. Fuel cell cars are finally reaching the marketplace in 2015; Toyota, Honda, and Hyundai are introducing models and have made commitments to build 100 hydrogen refueling stations in Japan and 48 in southern California by 2016 [86–88]. While these initial commitments represent a relatively small volume of hydrogen, gearing up to supply growing demand with solar-derived hydrogen, rather than hydrogen from steam methane reforming, would be a good exercise in developing the needed infrastructure for hydrogen generation, distribution, and storage. More generally, the issues associated with hydrocarbon-based transportation fuels are difficult to address with a carbon–neutral solution, as discussed above, until power plant operators face incentives to capture CO_2_ and convert it to fuels. In the short term, one could consider thermochemical pathways for generating more fuel per ton of biomass via pyrolysis and gasification pathways, both of which require hydrogen that solar could provide.

If solar fuels are to have a major impact on the energy supply mix in the long term, substantial research funding is needed to support innovations in the materials and technologies that underpin the solar refinery concept for delivering solar fuels. To identify promising technologies that warrant further research and development, it is useful to refer back to Table 1 prepared by Herron et al. [1]. Outside the thermochemical approach, a common theme in solar fuels production is the use of electrochemical cells, where the driving source is either solar electricity or direct photo-generated electrical current, as in photo-electrochemical cells. In general, the most critical opportunities for improvement are in *(photo)electrocatalysts*—specifically, improving efficiency (lower over-potentials), lowering costs (reduced use of noble metals), and extending life. Among commercial-scale electrolyzers, the PEM electrolyzer shows particular promise as the *power plant* when run in fuel cell mode in vehicles or as the companion to PV cells to generate hydrogen in the electrolysis mode. R&D funds directed toward identifying less costly and higher-temperature operating polymer electrolyte membranes as well as less costly and longer-lived oxygen evolution catalysts would both promote solar hydrogen production and solar energy storage by enabling integrated PV–hydrogen utility energy systems as illustrated in Fig. 2, as well as hydrogen use in fuel cell vehicles. Another technology that is currently less advanced, but that promises even higher efficiencies and the ability to co-electrolyze water and CO_2_ without the need for noble catalysts, is the solid oxide electrolysis cell (SOEC). Key limitations of the SOEC, which can also operate in reverse as a fuel cell, include its use of costly refractory materials and its potential for more rapid degradation at elevated operating temperatures. Research to improve the electrocatalytic behavior of anodes for oxygen evolution should lead to better electricity-to-hydrogen conversion efficiency and extended life at higher current densities/higher hydrogen evolution fluxes.

PEC cells, because they combine the separate functions of PV and electrolysis cells into a single cell, offer many potential advantages including higher efficiency, simplified assembly, and lower cost materials. However, the more complex criteria that PEC photoelectrodes must satisfy (e.g., efficient light absorption and charge transfer, chemical stability, and low cost) translate into greater challenges in finding optimized materials. Computational materials science [89] that can to be applied to screen the enormous numbers of potential candidate materials, together with high-throughput combinatorial fabrication methods [90], promises to rapidly identify promising alternatives. As in the PV field, tandem configurations offer potential for absorbing a greater fraction of incident radiation while also driving anodic and cathodic reactions at higher potentials. This would further enhance efficiency [57]. These and other efforts directed to prototyping and scale-up are very much needed and are being pursued, for example, at the Caltech Joint Center for Artificial Photosynthesis [91], at NREL [92], and at the University of North Carolina Energy Frontier Research Center for Solar Fuels [93].

Synthetic solar-derived hydrocarbon commodities, such as methane, methanol, and ethanol, are essential for satisfying the need to replace fossil fuels in transportation, heating, and energy storage and as a source of feedstocks for the chemicals, pharmaceutical, and fertilizer industries. The present technologies for capturing CO_2_ from flue gases (e.g., gas absorption into solvents or onto sorbents, membrane permeation, cryogenic distillation) remain costly and impose significant energy penalties for CO_2_ stripping and sorbent regeneration. Identifying membranes with high selectivity and permeability remains a great challenge. Photocatalytic processes capable of removing CO_2_ and simultaneously converting it to marketable hydrocarbon products deserve attention [94].

The use of *solar thermal power*, or *CSP,* to drive high-temperature thermochemical reactors, offers potential for achieving high solar-to-fuel energy conversion efficiencies and competitive costs in the short-to-intermediate term. Efforts to identify effective materials, coupled with the optimum combination of desirable thermodynamic, kinetic, and stability traits, would benefit as well from the application of computational materials science tools that are capable of comparing thousands of materials couples in short order. Scaling solar reactors and reducing heat losses are essential to achieving efficient, long-lived, and cost-effective systems.

In summary, harnessing solar energy to produce solar fuels offers, over the long run, the opportunity to replace fossil fuels as the major source of energy and commodity chemicals while also providing a means for storing energy from the essential but inherently intermittent solar resource. To become competitive in the market place, the local and efficient collection of CO_2_ from power plants or other sources and the low-cost production of H_2_ from water by photo-assisted electrolysis or by thermochemical means must simultaneously be established. This will require concerted research and development efforts in a number of key areas including photovoltaics, electrolysis and fuel cells, catalysts, efficient CO_2_ collection, hydrogen storage and distribution, and synthetic fuel production from CO and H_2_ feedstocks. Only a joint and concerted effort by government, industry and academia will lead to measurable progress in this critical endeavor.

## Footnotes

1 One liter of gasoline delivers 35 MJ of energy. This compares with the global average per capita energy consumption of ~900 MJ/day. Alternatively, one can compare the energy stored per kg of weight or per liter of volume of the storage medium: gasoline (46 MJ/kg; 32.4 MJ/L), hydrogen-compressed (123 MJ/kg; 5.6 MJ/L), lithium ion battery (0.36–0.88 MJ/kg; 0.9–2.63 MJ/L, and lead acid battery (0.17 MJ/kg; 0.56 MJ/L).

2 A preliminary version of this report entitled *Solar to Fuels Conversion Technologies* was made available on the MIT Energy Initiative website as a working paper associated with the study entitled *The Future of Solar Energy available* at http://energy.mit.edu/research/future-solar-energy/.

3 For example by low-cost electricity to hydrogen conversion via electrolysis, as discussed later.

4 If wind, the other major intermittent source of electric power, is included, the contribution from renewable sources in Germany over this time period increases to 2.17% of the total.

5 Until an efficient means for storing hydrogen becomes widely available.

6 Higher Heating Value (HHV), evaluated at room temperature and 1 atm pressure.

7 Steam reforming reaction: CH_4_ + H_2_O ⟺ CO + 3H_2_ (followed by water gas shift reaction CO + H_2_O ⟺ CO_2_ + H_2_ to further increase H_2_ yield).

8 The energy content of a gallon of gasoline and a kilogram of hydrogen are approximately equal on a lower heating value basis; a kilogram of hydrogen is approximately equal to a gallon of gasoline equivalent (gge) on an energy content basis.

9 One metric ton (MT) = 1000 kg.
